# Musculoskeletal symptoms of the upper extremities and the neck: A cross-sectional study on prevalence and symptom-predicting factors at visual display terminal (VDT) workstations

**DOI:** 10.1186/1471-2474-9-96

**Published:** 2008-06-27

**Authors:** André Klussmann, Hansjuergen Gebhardt, Falk Liebers, Monika A Rieger

**Affiliations:** 1 Institute of Occupational Health, Safety and Ergonomics (ASER) e.V., Corneliusstrasse 31, D-42329 Wuppertal, Germany; 2 Federal Institute for Occupational Safety and Health (BAuA), Noeldnerstrasse 40-42, D-10317 Berlin, Germany; 3 Department of Occupational Health and Environmental Medicine, Institute of General Practice and Family Medicine, Faculty of Medicine, University of Witten/Herdecke, Alfred-Herrhausen-Strasse 50, D-58448 Witten, Germany; 4 Institute of Occupational and Social Medicine, University Hospital Tuebingen, Wilhelmstraße 27, D-72074 Tuebingen, Germany

## Abstract

**Background:**

The aim of this study was to determine the prevalence and the predictors of musculoskeletal symptoms in the upper extremities and neck at visual display terminal (VDT) workstations.

**Methods:**

In a cross-sectional study 1,065 employees working at VDT > 1 h/d completed a standardised questionnaire. Workstation conditions were documented in a standardised checklist, and a subgroup of 82 employees underwent a physical examination.

**Results:**

Using the Nordic Questionnaire, the 12-month prevalence of symptoms of the neck, shoulder region, hand/wrist, or elbow/lower arm was 55%, 38%, 21%, and 15% respectively. The duration of VDT work had a significant impact on the frequency of neck symptoms in employees performing such work > 6 h/d.

**Conclusion:**

With regard to musculoskeletal symptoms of the upper extremities, preventive measures at VDT workstations should be focused on neck and shoulder symptoms (e.g. ergonomic measures, breaks to avoid sitting over long periods).

## Background

Musculoskeletal symptoms or disorders in the upper extremities and neck among employees working at visual display terminal (VDT) workstations has been a topic in occupational health research for many years. Yet, current prevalence data are rare in Germany. As working conditions may play a major role for symptom prevalence, a cross sectional study was performed focussing on workstations representative of German conditions with regard to VDT workstations' ergonomics and tasks to be performed by the employees. The findings were discussed against the background literature. Reviewing the national and international literature on the topic "work at visual display terminals (VDT) and musculoskeletal symptoms" with the help of the medical database PUBMED (years 1990 – 2007) and the literature database of the Federal Institute for Occupational Safety and Health (years 1990 – 2007) revealed several international studies dealing with this topic. In recent years the results published internationally indicated that employees may suffer from an impairment of general well-being and/or symptoms or disorders of the upper extremities due to working conditions [1-4]. Various nomenclatures have been used to label and characterise such disorders: RSI – repetitive strain injury, OOS – occupational overuse syndrome, OCD – occupational cervicobrachial disorder, and CTD – cumulative trauma disorder. One term that is internationally widely accepted is UEMSD or "upper extremity musculoskeletal disorders" [1,5-7]. With reference to a SALTSA study [8], on which some parts of the present study are based, the term "work-related upper extremity musculoskeletal disorders" (WRUEMSD) shall be used whenever the symptoms can be traced to working conditions. Controversial discussions are ongoing regarding the extent and etiology of the problem as well as the work-related causes and the risks leading to the symptoms – in particular the work at visual display terminal (VDT) workstations. Repetitive movements and activities can pose a significant harm to physical well-being. Sorgatz [9] described a "neuroplastic RSI model" derived from observations and diagnoses. According to this model, highly frequent repetitive movements cause micro lesions that accumulate in the affected musculoskeletal structures and lead to movement-related pain. Regular office work at the computer (data entry and use of the mouse) is supposed to induce disorders of the upper extremities. Public and scientific discussion of VDT-related office work has intensified in Germany in recent years: in 2004 the German micro census revealed that computer-related work constituted a large part of the daily working routine for approximately 21 million people (59% of all those in paid work) [10]. According to the following list of literature, several published studies have shown that the VDT workstation is becoming a great contributor to musculoskeletal disorders (Table 1).

**Table 1:** Selected international literature regarding symptoms or disorders in employees working at VDT workstations, in chronological order.

**Author(s)**	**Study design**	**Sample**	**Working hours**	**Amount of VDT work**	**Results**
Eltayeb et al. 2007 [11] (Netherlands)	cross-sectional	264 computer workers	not indicated (n.i.)	not indicated (n.i.)	- Prevalence of musculoskeletal complaints: neck: 33%, shoulder: 31%, upper arm: 12%, elbow: 6%, lower arm: 8%, wrist: 8%, hand: 11% (complaints during the previous year that lasted at least one week). - Higher prevalence of musculoskeletal symptoms in women than in men.
Ye at al. 2007 [12] (Japan)	cross-sectional	2,327 VDT users	n.i.	mean: 19.3 days/month	- Age less than 40 years, not receiving breaks during VDT work, and the presence of eyestrain and musculoskeletal pain were significantly associated with poor general health status (high GHQ scores). - Using a VDT for more than 5 h/day was also marginally associated with high GHQ scores in women (p < 0.1). - In conclusion, the management of physical health as well as work duration is important for good general health status among VDT users.
Thomsen et al. 2007 [13] (Denmark)	case-control	18 cases (VDT-workers with symptoms); 20 controls (VDT-workers without symptoms)	cases: 47.0 h/week controls: 35.5 h/week	cases: 28.6 h/week controls: 23.9 h/week	- Computer users with forearm pain and moderate to severe palpation tenderness had diminished forearm extensor muscle fatigue response. - Additional studies are necessary to determine whether this result reflects an adaptive response to exposure without any pathophysiological significance, or represents a part of a causal pathway leading to pain.
Kubo et al. 2006 [14] (Japan)	cross-sectional	2,161 office workers	n.i.	n.i.	- Positive relationship between VDT work and sick building syndrome (SBS) in men. - Association between prevalence of SBS an duration of VDT work in women. - Positive relationship between use of VDT and general symptoms, eye symptoms, respiratory symptoms, and skin rash. - The authors suggested that extended hours of VDT use might be related to increased SBS symptoms. Moreover, psychosocial distress related to VDT work might mediate the relationship between VDT use and SBS symptoms in women.
Gerr et al. 2005 [15] (USA)	intervention study (3 years)	3 intervetion groups nA = 122 nB = 125 nC = 115	n.i.	n.i.	- No differences in risk of musculoskeletal symptoms were observed among participants randomly assigned to two workstation and postural interventions in comparison to participants who received no workstation or pos-tural intervention. - The study provides no empirical basis for recommendation of one posture versus another for prevention of musculoskeletal symptoms among computer users.
Juul-Kristensen & Jensen 2005 [16] (Denmark)	cohort study	3,361 office workers in 11 Danish companies	n.i.	n.i.	- Working as much as 75% of the work time at the computer increased the probability of musculoskeletal disorders in the neck/shoulder and elbow/hand. - The speed of work was a prognostic factor for symptoms in the lower back.
Lassen et al. 2004 [17] (Denmark)	cohort study	6,943 technical assistants and machine technicians with VDT	69.5% full time work	24.3 h/week	- Detailed examination of self-reported exposures showed that mouse and keyboard-related work time predicted elbow and wrist/hand pain from low exposure levels without a threshold effect. - Mouse and keyboard-related work time were no predicting factors for clinical conditions.
Kryger et al. 2003 [18] (Denmark)	cohort study	same sample as in the study of Lassen et al. [17]			- Intensive use of a mouse device and (to a lesser extent) keyboard usage, were the main risk factors for forearm pain. - The occurrence of clinical disorders was low, suggesting that computer use is not commonly associated with any severe occupational hazard to the forearm.
Sillanpää et al. 2003 [19] (Finland)	cross-sectional	office workers (n = 298), customer service workers (n = 238) and designers (n = 247)	n.i.	total sample < 2 h/day: 2.3%; 2–4 h/day: 15.3%; 4–6 h/day: 23.0% > 6 h/day: 47.4%	- For all the occupations combined, the 12-month prevalence of musculoskeletal symptoms in the neck, shoulders, elbows, lower arms and wrists, and fingers were 63, 24, 18, 35 and 16%, respectively. - The study indicated that musculoskeletal pain is common among computer workers in offices. - There was no strong association between the duration of computer work and pain or between the duration of mouse use and pain, but workers' perception of their workstation as being ergonomically poor was strongly associated with an increased prevalence of pain. - Authors advise that more consideration should be paid to the ergonomics of workstations, the placing of the mouse, the postures of the upper extremities and the handling of the mouse.
Gerr et al. 2002 [20]; Marcus et al. 2002 [21] (USA)	cohort study	632 individuals with more than 15 h computer work per week.	38 h/week	mean 28 h/week	- Musculoskeletal symptoms (MSS) and disorders (MSD) in neck/shoulder (N/S) or hand/arm (H/A) were common among computer users. - More than 50% of computer users reported MSS during the first year after starting a new job. - The duration of keying (hours/week) was associated with H/A symptoms and disorders. - The most common N/S MSD was somatic pain syndrome. - Gender, age, ethnicity, and prior history of N/S pain were associated with N/S MSS and MSD. Gender, prior history of H/A pain, prior computer use, and children at home were associated with either H/A MSS or MSD. - Authors suggested that the risk of musculoskeletal symptoms and musculoskeletal disorders may be reduced by encouraging specific seating postures.
Nakazawa et al. 2002 [22] (Japan)	cohort study	25,000 office workers	n.i.	21% < 1 h/day 29% 1–3 h/day 22% 5–5 h/day 28% > 5 h/day	- Physical symptoms became more severe with increased daily VDT use without a threshold value effect. - Mental and sleep-related disorders in workers using VDT for more than 5 h/day were significantly higher than in groups using VDT for > 1, 1–3, and 3–5 h/day. - Duration of daily VDT use was linearly related to physical symptoms, and was non-linearly related to mental and sleep-related symptoms with a threshold effect of 5 h/day.
Ariens et al. 2001 [4] (Netherlands)	cohort study	1,334 workers from 34 companies	mean: 39.2 h/week	n.i.	- Sitting at work for more than 95% of the working time seems to be a risk factor for neck pain. - There tends to be a correlation between neck flexion and neck pain.
Bode & Isfort 2001 [23] (Germany)	cross-sectional	1,002 office workers (VDT ≥ 3 h/day)	n.i.	mean: 5 h/day	24-month prevalence: induration of the neck and shoulder area: 62%, muscle pain in the arm: 24%, paresthesia in the arm: 16%, paresthesia in the fingers or pain in the hand: 13% and 12% respectively (multiple answers possible). - Only 1/3 reported no symptoms.
Hartmann & Guetschow 1999 [24] (Germany)	cross-sectional	205 female office workers	n.i.	n.i.	- Monthly prevalence of symptoms: neck: 40%, shoulders: 36% - Hand/arm disorders seemed not to be the main issue of VDT users.
Ertel et al. 1997 [25] (Germany)	cross-sectional	352 female office workers	mean: 8.4 h/day	mean: 5.45 h/day	- Prevalence of symptoms during and after work: shoulder & neck: 62.7%, back: 53.0%, head: 45.3% and hands/arms/legs: 24.2%.
Michaelis et al. 1997 [26] (Germany)	cross-sectional	1,720 office workers	n.i.	n.i.	- Point prevalence of musculoskeletal symptoms: 62%. - Point prevalence of symptoms in the thoracic/lumbar spine in employees working at desk: 23% to 58% (increasing with age). Working at VDT increased prevalence by an average of 8%. - Point prevalence of symptoms in the cervical region is 20% to 36% (increasing with age). The prevalence was significantly higher in employees working at VDT compared to employees working only at desks.
Bergqvist et al. 1995 [27,28] (Sweden)	cross-sectional	353 office workers	n.i.	n.i.	- No general differences between VDT and non-VDT users as to the occurrence of muscular problems. - Combination of specific VDT work situations (e.g. typing work, work with a VDT for more than 20 h/week) and the presence of moderating factors was associated with excess risks of suffering from muscular problems.
Schwaninger et al. 1991 [29] (Germany)	cross-sectional	2,722 office workers in different companies	n.i.	33% 1/4, 27% 2/4, 27% 3/4, 13% 4/4 of work time	- Prevalence of musculoskeletal symptoms: neck pain: 38%, back pain: 38%, pain in the shoulders: 32%, pain in the arms/hands: 11% (no indication of reference period). - Only 1/3 reported no symptoms. - The authors recommended an optimal ergonomic configuration of the work place, multifaceted tasks and regular breaks at the VDT workstation.

### Aim of this study

The aim of this study was to determine the prevalence of work-related symptoms of the upper extremities and neck in employees who regularly perform VDT work. The cross sectional study focussed on workstations representative of German conditions with regard to VDT workstations' ergonomics and tasks to be performed by the employees. By means of standardised questionnaires, working conditions and employees' symptoms were assessed in order to describe approaches for preventive measures.

## Methods

### Instruments

A short checklist was used to evaluate the VDT workstations, a standardised questionnaire for the employees' survey, and a standardised medical diagnostic tool was designed for the physical examination.

### Workstation checklist

The checklist was based on a German VDT questionnaire (BiFra), which has been used since 1995 to evaluate various VDT workstations throughout Germany [30,31]), and is also available in French and English [32]. The respective reference database currently offers information on n = 18,620 VDT workstations [33]. Data on the set-up of the VDT workstations in the present study were compared with the BiFra database in order to check the representativeness of the sample.

The checklist used in this survey contains 37 items regarding display (e.g. size, reflections; 7 items), keyboard/mouse (e.g. area in front of the keyboard or for mouse-movement; 6 items), desk and arrangement of the VDT and accessories (e.g. adjustability of height, space for legs; 7 items), chair (e.g. adjustability of height; possibility of changing working postures; 9 items), ambient and environmental conditions (e.g. lighting of office and desk; 8 items). The items are dichotomous (attribute is fulfilled or not) and were completed by five ergonomists trained before the study.

### Standardised questionnaire

The employees' questionnaire was based on the Nordic Questionnaire [34], parts of the Copenhagen Psychosocial Questionnaire (COPSOQ [35]), and questions depicting work at the VDT. In this study, the respective German versions of these questionnaires were used [36,37].

The questionnaire contained 112 items including sociodemographic factors (e.g. age, gender, years on the job, leisure time activities, smoking habits; 13 items), musculoskeletal symptoms (e.g. prevalence, disability; 58 items), questions about viewing (e.g. symptoms at the eyes, use of corrective lenses or glasses; 5 items), kind and extend of VDT work (e.g. daily proportion of typing, data entry, monitoring, job rotation; 8 items), general working conditions (e.g. time pressure, shift work, working posture; 15 items), psychosocial factors (e.g. job satisfaction, cognitive demands, influence of work; 23 items). Questions on musculoskeletal symptoms were mostly dichotomous, questions on VDT work tasks demanded the indication of percentage of the very task per day (e.g. typing) and were metric, questions about the amount of breaks during VDT work or job rotation were given with ordinal scales (from always to never). Ordinal scales were used in the questions about psychosocial factors as well. For further calculations, the items about psychosocial factors taken from the COPSOQ were transformed to metric scales (0 to 100) according to the instructions of the author [35].

### Medical diagnostic tool

The medical diagnostic tool for the physical examination was added to analyse the extent to which symptoms could be attributed to specific tentative medical diagnoses using the SALTSA study's list of standard diagnoses of musculoskeletal disorders [8]. The diagnostic tool consisted of a documentation and a reference sheet. The documentation sheet was separated into three parts. Part A was a general survey to document painful or symptomatic body regions. Part B dealt with specific examination techniques to be carried out if pain or symptoms in the specific regions were documented in part A. According to these results and with the help of a reference sheet, tentative diagnoses could be derived and assigned in a list of diagnoses in part C. The diagnostic tool including the reference sheet were developed following the procedure recommended in a SALTSA study [8].

### Sample

**Figure 1:**
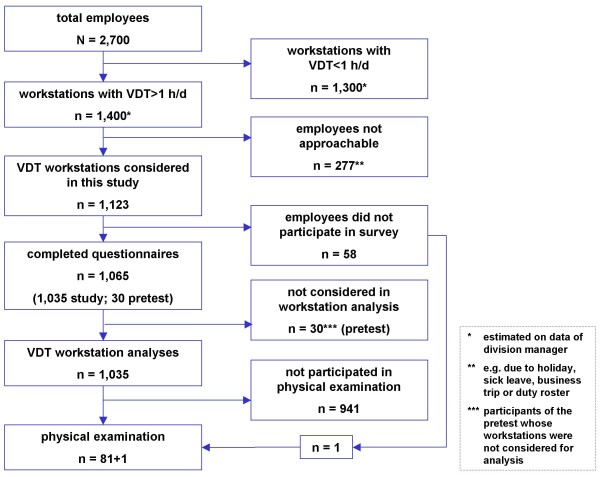
Study participants.

The study was carried out in 2005 at four sites of a large chemical company with a total of approximately 2,700 employees at that time. All employees performing VDT work for at least one hour per day were considered for the study. Those workstations at which the employees were absent on the assessment day (e.g. due to holiday, sick leave, business trip or duty roster) were not included into the study. A total of 1,123 VDT workstations were analysed with the help of the checklist. After obtaining informed consent, the employees were given the standardised questionnaire. 1,065 employees participated either by filling out the questionnaire on their own or were interviewed in a standardised way on the basis of the questionnaire. At the same time, the employees were informed about the additional physical examination, encouraging them to take advantage of this offer, in particular if they had any musculoskeletal symptoms. After the individual survey, the employees received advice on ergonomic and occupational safety and health issues and were informed about the possibility of physiotherapy. Eighty-two of the 1,123 employees underwent the physical examination offered on site at various times by an external medical doctor (Figure 1).

Of the 82 employees who took the physical examination, only 81 could be identified by means of an individual coding on the questionnaire. One employee did not complete the questionnaire, but nevertheless underwent the examination.

### Statistical methods employed

The aim of the survey was to determine various prevalences for musculoskeletal symptoms of the upper extremities, i.e. to get the percentage of subjects with symptoms in the relevant joints within a specific period. In addition, the 95% confidence interval (CI) was calculated. The calculation of a binomial confidence interval relies on approximating the binomial distribution with a normal distribution. According to Sachs [38], a binominal distribution is adequately approximated to normal distribution if n*p*q ≥ 9 (n = sample, p = frequency of outcome, q = 1-p).

The predictors of musculoskeletal symptoms were identified by means of logistic regression analysis and the calculation of odds ratios (OR) [39] using the SPSS^® ^12 statistical software, SPSS Inc. In all analyses alpha was set at p < 0.05 (two-tailed) [40].

**Figure 2:**
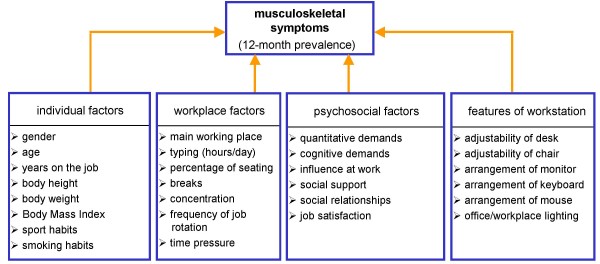
Factors with possible influence on symptoms.

According to existing literature on the topic, symptoms of the neck and the upper extremities may be caused by many factors [1,5-7,40-43]. Based on the generated data, multivariable analyses were conducted for the occurrence of symptoms in the various parts of the body, referring to all variables significantly associated with the presence of symptoms. The influence of personal, psychosocial, workplace, and work-related factors were calculated by means of logistical regression analysis for symptoms occurring in the neck, shoulder, elbow/forearm, and hand/wrist. Metric variables were used in the calculations (e.g. duration of daily VDT work or years on the job). The individual stress factors as well as moderating factors were derived from four categories: individual factors, workplace factors, psychosocial factors, and workstation characteristics (Figure 2). Yet they were only included into the model if no collinearity could be documented.

The analysis was carried out separately in six steps for the individual regions:

1. Initial check of variables to detect possible collinearity and determination of the remaining confounder.

2. Determination of the correlation of the individual factors and symptoms.

3. Determination of the workplace factors, adjusted for individual significant factors.

4. Determination of the psychosocial factors, adjusted for individual significant factors.

5. Determination of the features of the workstation, adjusted for individual significant factors.

6. Development of a final model considering only the factors ascertained as significant in step 2–5.

Variables were defined as collinear if the correlation between them was r > 0.4. The variable with the highest correlation with the outcome variable "symptom in this region" was retained in the model; the other variable(s) were deleted. Before forming the final model, logistic regression analyses was carried out for all the categories adjusted to the significant individual factors. Only the remaining significant variables were included in the final model.

### Ethics

The study was performed in compliance with the Helsinki Declaration. The aims, methods, and procedures of the study were coordinated with and agreed by the management and the workers' council of the company.

## Results

### Workstation characteristics

**Figure 3:**
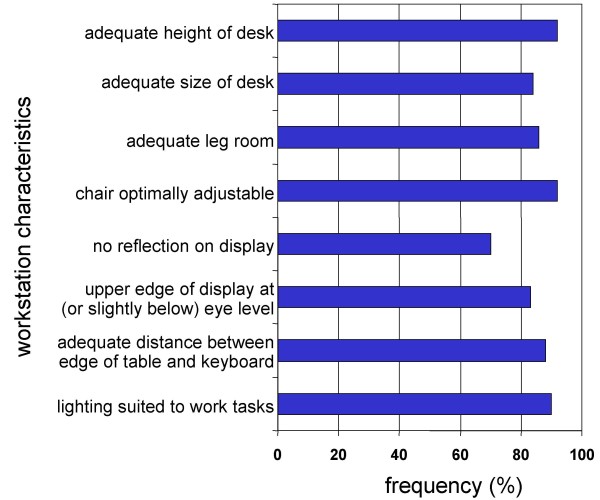
Characteristics of the evaluated VDT workstations; n = 1,035.

The VDT workstations were checked with regard to the ergonomic and spatial features given on the checklist (BiFra). Most of the workstations fulfilled the criteria of the checklist. Occasionally, reflections on the visual displays due to shortcomings in the lighting equipment were documented. With regard to the 1,035 workstations, for which the employees' questionnaires were also available, the most important ergonomic features of VDT workstations were fulfilled in 93% (height of desk) or 60% (absence of reflection on the display) of the workstations (Figure 3).

### Description of the employees' sample

Among the 1,065 employees participating in the survey, 803 performed only office work while 159 worked in laboratories and 82 in the areas of storehouse and production. 16 workstations, listed as miscellaneous, could not be allocated to one of the four groups (e.g. doormen). About 65% of the study participants were male. The mean age was 39.9 (± 9.5) years and the mean daily VDT use amounted to 5.1 (± 2.3) hours/day (Table 2).

**Table 2:** Characteristics of the employees' sample: gender, age, and duration of daily VDT use at various workplaces (> 1 h/d)

**Workplace**	**Gender**	Number* (n)	**Age**		**Daily VDT work**		**Percentage of all**
					
			**(years)**	**(± sd)**	**(hours)**	**(± sd)**	**(%)**
**Office**	Female	306	38.8	10.8	5.9	2.1	28.9
	Male	497	41.5	9.0	5.4	2.1	46.9
	**Total**	**803**	**40.5**	**9.5**	**5.6**	**2.1**	**75.8**
**Laboratory**	Female	65	37.6	8.9	4.0	2.2	6.1
	Male	94	39.0	9.7	3.1	1.6	8.9
	**Total**	**159**	**38.4**	**9.4**	**3.5**	**1.9**	**15.0**
**Storehouse/production**	Female	5	30.6	5.6	3.5	1.0	0.5
	Male	77	38.1	9.0	3.9	2.5	7.3
	**Total**	**82**	**37.6**	**9.0**	**3.9**	**2.5**	**7.7**
**Miscellaneous**	Female	5	28.8	9.4	3.1	2.0	0.5
	Male	11	41.4	11.2	5.2	3.8	1.0
	**Total**	**16**	**37.4**	**12.0**	**4.6**	**3.5**	**1.5**
**All**	Female	381	38.3	9.9	5.5	3.2	35.9
	Male	679	40.8	9.2	4.9	2.3	64.1
	**Total**	1,060*	**39.9**	**9.5**	**5.1**	**2.3**	**100.0**

* 5 subjects didn't indicate their age

### Symptom prevalence

By means of the Nordic Questionnaire, the lifetime, 12-month, 1-month, 1-week, and point prevalence of neck, shoulder, elbow/forearm, and hand/wrist symptoms was determined. With regard to the 12-month prevalence of the whole sample, the highest values were described in the neck (55%) and shoulder region (38%). The least pronounced occurrences were seen in the hand/wrist and elbow/forearm with values of 21% and 15%, respectively (bottom row in Table 3). Similarly, the 1-week prevalence was highest in the neck (21%) and shoulder region (15%), and lowest in the hand/wrist (7%) and elbow/forearm (5%, Table 4). In most of the symptom classes, women showed higher prevalence than men.

**Table 3:** 12-month symptom prevalence (bold font) with 95% confidence interval (95%-CI) and total number of affected employees classified according to age groups. (prev.=prevalence)

**12-month prevalence**			**Symptoms of neck**			**Symptoms of shoulder**			**Symptoms of elbow/forearm**			**Symptoms of hand/wrist**		
						
			**Prev. (%)**	95%-CI	n	**Prev.(%)**	95%-CI	n	**Prev. (%)**	95%-CI	n	**Prev. (%)**	95%-CI	n
Female, n = 379	Total female		**66.0**	61.2, 70.7	250	**45.6**	40.6, 50.7	173	**15.6**	11.9, 19.2	59	**24.5**	20.2, 28.9	93
	Age group	< 30	**63.6**	53.6, 73.7	56	**39.8**	29.5, 50.0	35	**12.5**	5.6, 19.4	11	**28.4**	19.0, 37.8	25
		30–39	**68.9**	60.6, 77.2	82	**41.2**	32.3, 50.0	49	**12.6**	6.6, 18.6	15	**23.5**	15.9, 31.2	28
		40–49	**67.3**	58.4, 76.2	72	**50.5**	41.0, 59.9	54	**18.7**	11.3, 26.1	20	**20.6**	12.9, 28.2	22
		50–59	**62.5**	50.6, 74.4	40	**54.7**	42.5, 66.9	35	**20.3**	10.4, 30.2	13	**28.1**	17.1, 39.1	18

Male, n = 678	Total male		**48.1**	44.3, 51.9	326	**32.8**	29.3, 36.3	223	**14.8**	12.1, 17.5	100	**18.9**	16.0, 21.8	128
	Age group	< 30	**41.3**	30.5, 52.1	33	**20.0**	11.2, 28.8	16	**11.3**	4.4, 18.2	9	**8.8**	2.6, 15.0	7
		30–39	**49.2**	42.9, 55.5	120	**30.7**	24.9, 36.5	75	**11.1**	7.2, 15.0	27	**20.1**	15.1, 25.1	49
		40–49	**48.6**	42.0, 55.2	107	**36.4**	30.0, 42.8	80	**16.8**	11.9, 21.7	37	**19.5**	14.3, 24.7	43
		50–59	**51.6**	42.9, 60.3	66	**40.6**	32.1, 49.1	52	**20.1**	13.2, 27.0	27	**22.7**	15.4, 30.0	29

All n = 1,057			**54.5**	51.5, 57.5	576	**37.5**	34.6, 40.4	396	**15.0**	12.8, 17.2	159	**20.9**	18.4, 23.4	221

**Table 4:** 1-week symptom prevalence (bold font) with 95% confidence interval (95%-Cl) and total number of affected employees classified according to age groups. (prev.=prevalence)

**1-week prevalence**			**Symptoms of neck**			**Symptoms of shoulder**			**Symptoms of elbow/forearm**			**Symptoms of hand/wrist**		
						
			**Prev. (%)**	95%-CI	n	**Prev. (%)**	95%-CI	n	**Prev. (%)**	95%-CI	n	**Prev. (%)**	95%-CI	n
Female, n = 379	Total female		**28.0**	23.4, 32.5	106	**19.3**	15.3, 23.2	73	**4.0**	2.0, 5.9	15	**7.9**	5.2, 10.6	30
	Age group	< 30	**30.7**	21.0, 40.3	27	**17.0**	9.2, 24.9	15	**1.1**	*	1	**4.5**	*	4
		30–39	**27.7**	19.7, 35.8	33	**15.1**	8.7, 21.5	18	**3.4**	*	4	**7.6**	*	9
		40–49	**29.0**	20.4, 37.6	31	**22.4**	14.5, 30.3	24	**3.3**	*	7	**9.3**	3.8, 14.9	10
		50–59	**23.1**	12.8, 33.3	15	**24.6**	14.1, 35.1	16	**4.6**	*	3	**10.8**	*	7

Male, n = 678	Total male		**16.4**	13.5, 19.0	111	**13.1**	10.5, 15.5	89	**5.5**	3.7, 7.1	37	**5.9**	4.1, 7.6	40
	Age group	< 30	**16.3**	8.2, 24.3	13	**8.8**	*	7	**1.3**	*	1	**2.5**	*	2
		30–39	**12.7**	8.5, 16.9	31	**9.8**	6.1, 13.5	24	**3.3**	*	8	**5.3**	2.5, 8.1	13
		40–49	**13.2**	8.4, 17.7	29	**12.7**	8.0, 17.5	28	**5.9**	2.6, 9.3	13	**5.9**	2.6, 9.3	13
		50–59	**28.4**	20.8, 36.0	38	**22.4**	15.3, 29.5	30	**11.2**	5.9, 16.5	15	**9.0**	4.2, 13.7	12

All n = 1,057			**20.5**	18.0, 22.8	217	**15.3**	13.0, 17.4	162	**4.9**	3.6, 6.2	52	**6.6**	5.1, 8.1	70

* due to the small prevalence, a 95%-CI could not be calculated [38]

### Identification of symptom-predicting factors

Symptom-predicting factors were identified with the help of the six steps of multivariable analyses on the basis of the 12-month prevalence, as described above. Despite analysing separately for individual regions (neck, shoulder, elbow/forearm, hand/wrist), the results of these steps were described cohesively as some predictors are the same. First, the initial variables (Figure 2) were checked for collinearity (step 1).

Analysing the individual factors (step 2) revealed significant effects of gender on the neck (odds ratio (OR): 2.02, p < 0.001) and shoulder regions (OR: 1.83, p < 0.001), of years on the job on the shoulder region (OR: 1.02, p < 0.001) and elbow/forearm (OR: 1.02, p < 0.05), and of the Body Mass Index (BMI) on the elbow/forearm (OR: 1.05, p < 0.05). Sports and smoking habits had no significant effects on symptoms in any body region. Body height and weight were deleted as they correlated strongly with the BMI but the correlation between BMI and symptoms was higher. Also age was deleted as it correlated strongly with years on the job but the correlation between years on the job and symptoms was higher.

In step 3 (workplace factors adjusted to significant individual factors), typing (OR: 1.10, p < 0.01) and the frequency of job rotation (OR: 1.91, p < 0.001) showed a significant effect on neck symptoms, and working in laboratory compared to office workstations revealed a low significant effect on symptoms of the elbow/forearm (OR: 2.00, p < 0.05). Sitting was eliminated as it correlated strongly with typing but the correlation between typing and symptoms was higher. Brain work over long time periods and time pressure had no significant effects on symptoms in any body region.

Investigating the psychosocial factors (step 4, adjusted to significant individual factors) showed only one factor with significant effect (job satisfaction – symptoms of elbow/forearm: OR: 0.98, p < 0.01).

Quantitative demands, cognitive demands, influence at work, social support from colleagues and superiors, and social relationship in the company had no significant effects on symptoms in any body region.

In step 5 (features of the workstation adjusted to significant individual factors) adjustability of the chair showed a low significant effect on symptoms in the elbow/forearm (OR: 0.52, p < 0.05). Other factors (adjustability of desk, arrangement of monitor, keyboard and mouse or lighting of the office/workstation) had no significant effects on symptoms in any body region.

In the final model (step 6), all factors with significant effects in the above steps were analysed with respect to every individual region (Table 5). Gender, typing, job rotation, and job satisfaction showed significant effects with regard to symptoms of the neck. Symptoms of the shoulder region were significantly predicted by the factors years on the job and gender. The duration of work also significantly influenced the prevalence of symptoms of the elbow/forearm region, which were also modified by Body Mass Index and job satisfaction. The frequency of job rotation was the only factor which influenced the prevalence of symptoms of the hand/wrist. The explained variance of the final models described above was very small and resulted in an explanatory power of Nagelkerke's R-square with 11% for the neck, 5% for the shoulder, 4% for the elbow/forearm, and 3% for the hand/wrist model.

**Table 5:** Multivariable analysis of symptom-predicting factors (12-month prevalence) – final model (step 6). The bold font indicates the significant factors.

		**Symptoms of neck**			**Symptoms of shoulder**			**Symptoms of elbow/forearm**			**Symptoms of hand/wrist**		
				
**Significant factors **		p-value	OR	95%-CI	p-value	OR	95%-CI	p-value	OR	95%-CI	p-value	OR	95%-CI
Years on the job		0.255	1.008	0.994, 1.022	**0.000**	**1.026**	**1.012, 1.040**	**0.008**	**1.025**	**1.006, 1.043**	0.399	1.007	0.991, 1.023
Gender		**0.000**	**2.005**	**1.478, 2.719**	**0.000**	**1.890**	**1.399, 2.553**	0.285	1.247	0.832, 1.868	0.069	1.385	0.975, 1.967
Body Mass Index		0.196	1.027	0.987, 1.068	0.261	1.023	0.983, 1.064	**0.027**	**1.058**	**1.007, 1.113**	0.303	1.024	0.979, 1.072
Main work-place	Office	0.424			0.787			0.572			0.272		
	Laboratory	0.140	1.345	0.907, 1.995	0.506	1.145	0.769, 1.704	0.167	1.438	0.860, 2.404	0.071	1.523	0.965, 2.403
	Production/storehouse	0.823	0.942	0.559, 1.587	0.663	1.128	0.656, 1.940	0.572	1.235	0.594, 2.566	0.679	0.861	0.423, 1.751
	Other	0.516	1.519	0.430, 5.361	0.427	1.621	0.492, 5.337	0.768	1.245	0.289, 5.363	0.920	1.074	0.267, 4.316
Typing		**0.012**	**1.096**	**1.020, 1.177**	0.228	1.045	0.973, 1.122	0.062	1.096	0.995, 1.207	0.276	1.048	0.964, 1.139
Job rotation		**0.001**	**1.740**	**1.255, 2.410**	0.244	1.211	0.877, 1.673	0.997	0.999	0.641, 1.557	**0.020**	**1.558**	**1.073, 2.263**
Job satisfaction		**0.002**	**0.986**	**0.977, 0.995**	0.362	0.996	0.987, 1.005	**0.013**	**0.985**	**0.974, 0.997**	0.392	0.995	0.985, 1.006
Chair optimally adjustable		0.615	0.860	0.478, 1.548	0.481	0.810	0.451, 1.456	0.202	0.631	0.311, 1.280	0.064	0.551	0.293, 1.036

Constant		0.598	0.698		0.018	0.203		0.001	0.060		0.025	0.175	

**Figure 4:**
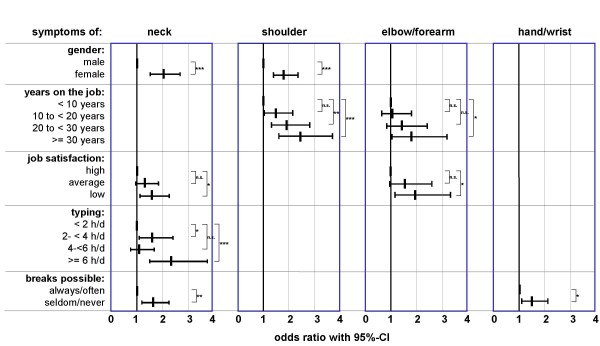
Predictors for the 12-month symptom prevalence – final model: odds ratio with 95% confidence interval.

For the purpose of illustration, the factors showing a significant association to the symptoms in the final models were gathered in Figure 4. Here, the metric factors were categorised into groups.

### Results of the physical examination

A total of 82 employees took advantage of the offer to undergo a physical examination; 37 female (mean age: 41.8+/-8.8 years) and 45 male subjects (mean age: 45.0 +/-8.7 years). In comparison with the total sample, this subgroup was, on the average, 3.7 years older (significant difference p < 0.001, ANOVA) and the percentage of women was higher (45% vs. 36% in the total sample; significant difference p < 0.05, CHI^2^). In general, subjects who underwent the physical check suffered more frequently from acute pain than employees who did not take the examination (significant difference p < 0.001, CHI^2^) (Table 6).

**Table 6:** Characteristics of the subgroup who took the medical examination compared to the total sample

	**n**	**Gender**	**Age (years)**	**Daily VDT work (hours)**	**Working hours per week**	**Actual symptoms (%)**			
						
						Neck	Shoulder	Elbow/forearm	Hand/wrist
**Total sample **	1,065	684 men	39.9 (**± **9.5)	5.1 (**± **2.3)	40.9 (**± **6.8)	14.5	11.5	3.5	4.5
**Subgroup**	82	45 men	43.6 (**± **8.4)	5.8 (**± **2.2)	40.6 (**± **6.0)	43.9	31.7	13.4	13.4

**Figure 5:**
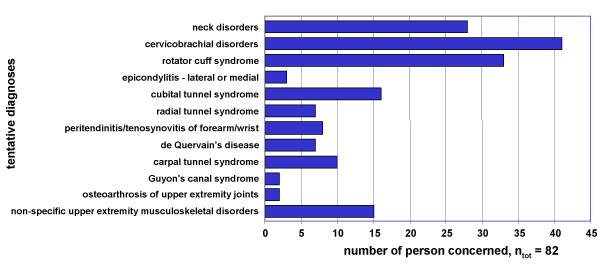
Diagnoses among the employees who underwent physical examination (n = 82) (multiple answers were possible).

As suggested by the SALTSA study mentioned above, during the physical examination the tentative diagnoses were made even when symptoms occurred only in a mild form. Disorders of the cervicobrachial and neck region and rotator cuff syndrome were diagnosed most frequently (Figure 5). Tentative diagnoses were found slightly more often in women than in men, but because of the small number of participants, these differences were not significant.

## Discussion

### Aims of the study and review of methods

The basic aim of the study was to describe the period prevalence of musculoskeletal symptoms in the upper extremities among employees working at VDT workstations representative of German office workstations. In addition, factors predicting the occurrence of symptoms were to be identified. Both aims were achieved.

With the help of a standardised questionnaire and a checklist, the main features of VDT workstations were described, the employees were interviewed regarding their work and possible symptoms, and standardised physical examinations were carried out. The high degree of participation (95% of the subjects addressed) suggested that the employees were very interested in the topic and that the questionnaire was easily to complete. Whereas the high proportion of survey participants may be related to the frequent occurrence of musculoskeletal symptoms in VDT workers, the symptoms were apparently not severe enough to motivate the employees to seek medical advice. The checklist for evaluating the VDT workstations (based on BiFra [30,31]), the employees' questionnaire (based on the Nordic Questionnaire [34] and the COPSOQ [35]), and the physical examination tool kit (based on the SALTSA Study [8]) were well suited for the study and can be recommended for further studies.

### Representativeness of the working conditions

**Figure 6:**
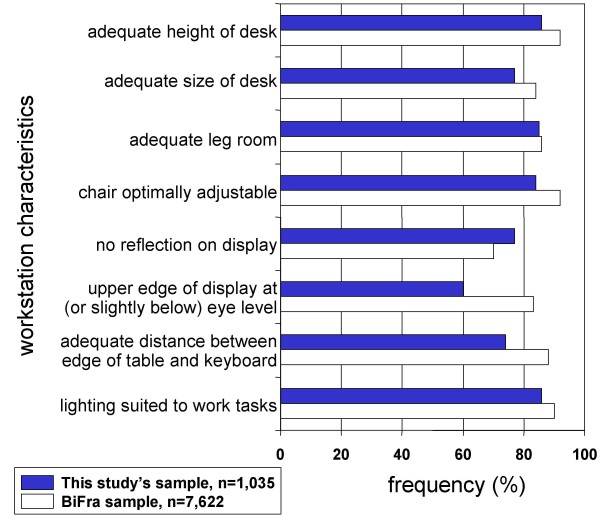
Results of workstation evaluation and data of the BiFra database (2000–2005).

Generally, the ergonomic conditions at the investigated workstations can be considered as good or very good. The majority of the working places fulfilled all criteria of the checklist. The only deficiencies regarding the setup of the workplace were detected at VDT workstations where employees only worked for a limited time, such as production or storage areas. As illustrated by the BiFra database, a high ergonomic quality of the workstation can apparently be found in a variety of other industries and services in Germany as well. The comparative presentation of workstation evaluations from the years 2000 to 2005 (n = 7,622), extracted from the BiFra database, and of the results of the presented study revealed the good representativeness of working conditions investigated in the survey (Figure 6). Yet it has to be borne in mind that the good equipment of the workstations influences the observed prevalence of symptoms as well as the predictors derived from the multivariable analyses.

### Symptom prevalence

As with the 12-month symptom prevalence, the 1-week prevalence results revealed that the neck and shoulder symptoms were clearly more prevalent than the hand/wrist and elbow/forearm symptoms. With regard to the 12-month prevalence of the whole sample, the highest values were found in the neck (55%) and shoulder (38%) region. The least pronounced prevalence was found in the hand/wrist and elbow/forearm, with values of 21% and 15%, respectively. These results are similar to the findings of a study among computer office workers from the Netherlands using the Maastricht Upper Extremity Questionnaire (MUEQ). In this study, similarly to our findings, the most commonly reported complaints were neck and shoulder symptoms (33% and 31%, respectively), followed by upper arm complaints and hand (12% and 11%), and lower arm, wrist, and elbow complaints (8%, 8%, and 6%) [11]. Comparing the figures, it is important to note that the MUEQ addressed symptoms only if they lasted at least one week during the previous year – thus being different from the Nordic Questionnaire. This fact might explain the considerable differences in the prevalence between the German and Dutch VDT-workers whereas the hierarchy of symptoms was the same in both studies. In a Finnish sample consisting of office workers, customer service workers, and designers, the 12-month prevalence of musculoskeletal symptoms were 63% in the neck, 24% in the shoulders, 18% in the elbows, 35% in the lower arms and wrists, and 16% in the fingers [19]. This is a higher prevalence of neck symptoms and symptoms of the lower arms and wrists than in the present study. This might be explained by the higher proportion of women in the Finnish survey.

**Figure 7:**
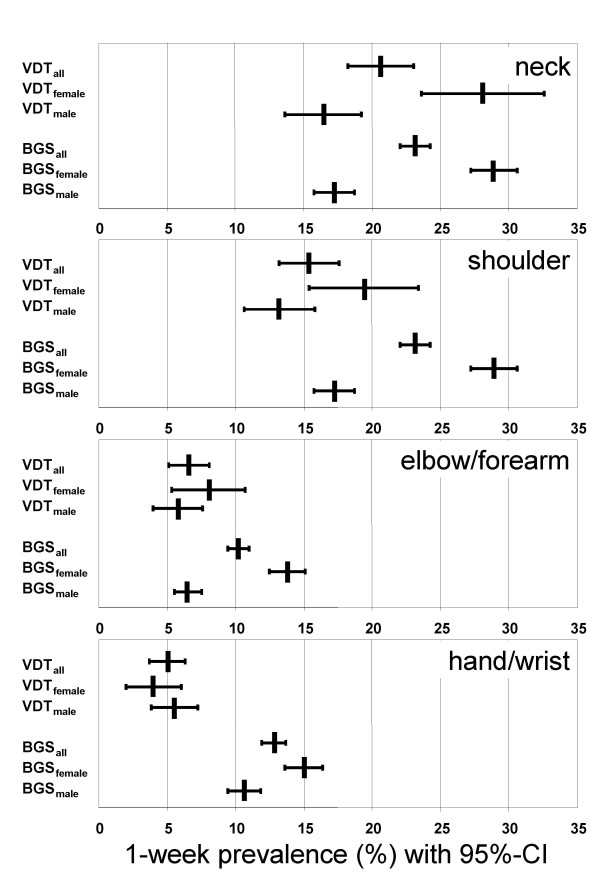
1-week prevalence of symptoms with 95% confidence interval (95%-CI). VDT workers (n = 1,065) (VDT) vs. random sample of German population derived from the German Health Survey (BGS) (extract: 20 to 60 years old, n = 5,208).

The prevalence described in the present study can also be qualified by comparing it with the data of the German Health Survey 1998 (BGS'98 [44]), where the 1-week prevalence of specific symptoms was analysed. The data from the BGS'98 were derived from interviews with 7,124 subjects who were arbitrarily selected and who, according to the authors, can be considered representative of the German population. The comparison of both studies is, however, subject to two qualitative restrictions: The BGS study took place 6–7 years before the survey presented, for which reason a bias in the results of the comparison cannot be excluded. Furthermore, the BGS had a response rate of only 61.4%, which may represent another possible bias risk. From the BGS, data of the 5,208 subjects aged between 20 and 60 years were extracted in order to perform an age-related comparison to the results of the present study (Figure 7). The BGS study group consisted of 2,644 women (mean age 41.0 ± 11.4 years) and 2,564 men (mean age 40.8 ± 11.5 years), thus revealing a similar age distribution (mean age 40.9 ± 11.4 years) to the study's participants (mean age 39.9 ± 9.5 years).

Shoulder, elbow/forearm, and hand/wrist symptoms are significantly more common in the BGS group than in the group of VDT workers (Figure 7). These differences are particularly substantial in women.

These findings should not be interpreted in a way that suggests that VDT work has a protective effect on symptoms of the shoulder, elbow/forearm, or hand/wrist. Instead, the authors suppose that the participants of the present study indicated less symptoms as they were thinking mainly on work-related symptoms (negative reporting bias).

### Predictors

Based on the generated data, multivariable analyses were conducted for the occurrence of symptoms in the various parts of the body, identifying an optimal set of variables explaining a maximum part of the variance in the presence of symptoms. As a result of this final analysis, only a few predictors could be identified for musculoskeletal symptoms of the upper extremities in the present study focussing on employees working at VDT workstations for more than 1 hour per day. Among the multitude of possible influencing factors investigated, only more than 20 years on the job, a high lack of job satisfaction, typing for at least 6 h/d, and limitations to take breaks significantly increased the 12-month prevalence of one or more musculoskeletal symptoms. In addition, women indicated symptoms in the neck and shoulders more frequently than men.

Most of the studies of work-related musculoskeletal symptoms or disorders reported a higher prevalence of risk in women than in men, regardless of the kind of work or occupation involved. The same difference exists between woman and men regarding VDT users [11,17,20,21,41-43]. For more details see the review by Wahlstroem [43].

Ekman et al. suggested that the higher prevalence of symptoms in women may be due to non work-related factors or that there could be a difference in the occupational exposure among men and women [42]. In a review [46], possible reasons were summarised in the following four groups:

- differences in task type allocations or work tasks between men and women,

- higher physical stress or stress load of women from non-work activities such as childcare and household work,

- physiological differences, such as different body size or body mass or endocrine functions, and

- differences in the willingness to report or seek medical care for pain or discomfort.

Gerr et al. observed that women had higher values in reporting symptoms and were also at increased risk for disorders confirmed by physical examination [20]. These findings confirm our observation that more women than men took the advantage of the physical examination. Yet in the present study the prevalence of disorders confirmed by physical examination did not differ significantly between men and women. To sum up, there seems to be evidence for women's increased risk of musculoskeletal disorders [20], but more research is needed on this topic.

Psychosocial factors have been discussed as predictors in many previous studies [4,41,46]. In a review, high job demands, low decision latitude, time pressure, mental stress, job dissatisfaction, high workload, and lack of social support from colleagues and superiors were suggested as risk factors for musculoskeletal disorders in computer workers [43].

In our model we used scales taken from the COPSOQ [35] to consider psychosocial factors concentrating on quantitative demands, cognitive demands, influence at work, social support from colleagues and superiors, social relationship in the company, and job satisfaction. Interestingly enough there was only a low but significant relationship between job satisfaction on the one hand and neck and hand/wrist symptoms on the other. Thus our findings correspond partly to the results of Ariens et al. [4] who described psychosocial factors as independent risk factors for neck pain.

With regard to work organisation, large amounts of typing and limited breaks during VDT work have been described as risk factors for musculoskeletal symptoms. In the present study "typing" more than 6 hours per day at a VDT workstation had a significant impact on the prevalence of neck symptoms. A similar exposure ("keying") was reported by Gerr et al. to be positively associated with hand/arm symptoms and disorders [20,21]. In two Japanese studies, effects of the duration of daily VDT work on physical symptoms [22] or – in women – on the general health status [12] were documented. Bergqvist et al. described that combinations of specific VDT work situations (e.g. typing work, work with a VDT for more than 20 h/week) together with moderating factors were associated with an excess risk of suffering from muscular problems [27,28]. The importance of the amount of VDT work was documented by Juul-Kristensen & Jensen [12] as well. These authors found that working as much as 75% of the working time at a computer increased the probability of musculoskeletal disorders in the neck/shoulder and elbow/hand.

However it seems to be questionable whether the VDT work as such or other aspects of computer work are related to the symptoms. According to Ariens et al. [4], sitting at work for more than 95% of the working time seems to be a risk factor for neck pain. In the present study sitting was strongly associated with typing and the amount of VDT work. Because of the close correlation between sitting, typing, and VDT work, there is no clear evidence as to which of these factors is the main predictor for neck (or other) musculoskeletal symptoms.

In the scientific literature there seems to be a consensus on poor ergonomic conditions at VDT workstations contributing to musculoskeletal symptoms or disorders [22,43,46]. As mentioned above, the majority of the workstations considered in this study were well or very well equipped. Due to this high ergonomic standard and the small variance found in our sample, the workstation characteristics had no effects on symptom prevalence in the multivariable analysis. A similar effect was reported by Michaelis et al. with respect to the possible impact of ergonomic factors on back pain [26].

In the sample investigated, some predictors could be identified for musculoskeletal symptoms of the upper extremities. Due to the stepwise procedure applied in the multivariable analyses, collinear factors could be excluded thus leading to rather slender models for the symptoms in the different regions. Yet it must be borne in mind that most of the ORs, and their lower confidence limits, are very close to unity and that the explained variance in the models was only small (Nagelkerke's R-square: 3–11%).

### Physical examination

The figures of employees who voluntarily participated in the physical examination suggests that approximately 8% of the total sample could be addressed with intensive campaigning. The majority of the employees seeking medical advice seemed to do so because of acute or chronic pain.

The symptoms expressed by the employees could mostly be confirmed in the physical examination; tentative diagnoses were made for these cases. The diagnoses also revealed the great importance of symptoms in the shoulder and neck region as the most frequently diagnoses were cervicobrachial disorders, neck disorders, and the rotator cuff syndrome.

### Significance of the results

Generally speaking, this study confirms the main findings of the literature [4,11-29]. Neck and shoulder symptoms occurred significantly more often than symptoms in the distal parts of the upper extremities. Neck symptoms were associated positively with a large amount of typing per day. The data referring to prevalence, gender, age distribution, and duration of daily VDT work in the various studies can be considered comparable despite the fact that the questionnaires differ somewhat. For these reasons the results achieved here can be considered representative. The significant new findings of the current study are based on the simultaneous consideration of various regions of the upper extremities, various time periods (e.g. 1-week, 12-month prevalence), as well as the reporting of confidence intervals.

### Limitations of this study

Information about workplaces and VDT workstations was obtained by ergonomists and can be considered objective. In contrast the information about musculoskeletal symptoms, psychosocial factors, and amount of daily VDT work was obtained by a survey of the employees thus being prone to over- or underestimation. The difference between self-reporting and observation of others in physical work was recently assessed to reach between 30–45% [47,48].

The aim of this study was to determine the prevalence of symptoms of the upper extremities and neck and to describe possible predictors derived from working conditions. Information about non-occupational stress factors was not assessed, e.g. children at home, household work, ethnicity, or the history of symptoms described as predictors in literature [20]. In addition, the factor work style was not assessed, i.e. the strategy that workers may employ for completing, responding to, or coping with job demands that might affect musculoskeletal health [49,50].

The possible impact of these factors might account for the fact that the explained variance was only low (Nagelkerke's R-square: 3–11%) in the study presented.

Generally, the study is limited by the cross-sectional design, which is not suitable to assess the causal relationship between variables but only associations.

With regard to sample size and the high response rate (95%), a "healthy worker effect" among the employees addressed has to be considered. In addition, no information was available about the prevalence of musculoskeletal complaints in the small group of 277 employees absent on the assessment day. Sick leave due to musculoskeletal symptoms could be increased in this group. Both factors would lead to a minor underestimation of prevalence in the present study. Nevertheless, the overall response was high; therefore response bias seems to be unimportant.

### Preventive measures

The majority of the workstations fulfilled all criteria of the checklist with exception of those workstations that tended to be used irregularly and temporarily, i.e. workstations in the production or storage areas. Thus, the employees had rather good ergonomic conditions. Yet, the symptom prevalence at the VDT workstations investigated in the present study was impressive. The multivariable analyses showed that neck and hand/wrist symptoms occurred less frequently when VDT work was interrupted periodically and other tasks were performed. It is therefore recommendable that office employees vary their tasks regularly – even if the workstation guarantees a high ergonomic standard. In addition, physiotherapeutic measures can be employed to counteract the occurrence or aggravation of tissue injury. Furthermore, employee motivation and the involvement of employees in decision-making processes are measures that may increase job satisfaction and, in doing so, can have a positive impact on the physical as well as mental well-being of the employees.

## Conclusion

With regard to musculoskeletal symptoms, preventive measures should focus on neck and shoulder disorders. As derived from this study, work organisation plays an important role, especially when ergonomic measures are largely implemented. The organisation of work should allow regular breaks of VDT work and avoid large amounts of continuous typing. The methods used in this study proved suitable to assess the workstation characteristics and the employees' symptoms thus helping to derive appropriate measures to avoid or moderate physical impairment. The data gathered in this survey can be used as a reference for further studies with comparable outcomes and in occupational safety and health campaigns addressing the ergonomic characteristics of VDT workstations. Occupational health services will be able to use the diagnostic tool kit for physical examination thus checking for tentative diagnoses which may be confirmed by a medical specialist and, if appropriate, via apparative diagnostics.

## Competing interests

The authors declare that they have no competing interests.

## Authors' contributions

AK, HG, and MAR conceived and carried out the study and drafted the manuscript. FL represents the funding body, initiated this study, and was closely involved in the planning and development of the study design. All authors read and approved the final manuscript.

## Pre-publication history

The pre-publication history for this paper can be accessed here:


